# Doing a double take: when transthoracic echocardiography fails for mitral valve annuloplasty ring endocarditis with *Streptococcus pyogenes*: a case report

**DOI:** 10.1186/s13256-024-04623-y

**Published:** 2024-07-13

**Authors:** Jonathan K. Schilling, Robert Tungate, Pranav M. Patel, Jack Sun, Antonio H. Frangieh

**Affiliations:** 1 Department of Medicine, University of California, Irvine, 333 City Blvd. West Suite 400, Orange, CA USA; 2 Department of Cardiology, University of California, Irvine, Orange, CA USA; 3 Department of Surgery, University of California, Irvine, Orange, CA USA

**Keywords:** Culture-negative endocarditis, *Streptococcus pyogenes*, Mitral valve annuloplasty, Prosthesis, Case report

## Abstract

**Background:**

This case highlights several complications of a late and rare presentation of culture-negative *Streptococcus pyogenes* endocarditis of a previously repaired mitral valve with an annuloplasty ring including recurrent cardioembolic strokes, which was initially missed on transthoracic echocardiography.

**Case presentation:**

A 66-year-old Caucasian female with prior mitral valve prolapse status post mitral valve annuloplasty and left atrial appendage occlusion, followed by two strokes, presented with supraventricular tachycardia that resolved spontaneously. During an inpatient admission, she developed symptoms of another stroke, and imaging studies were suggestive of recurrent cardioembolic phenomenon. Additional workup revealed two small intra-atrial masses adherent to the mitral annuloplasty ring missed on prior evaluation for recurrent stroke. She underwent surgical repair in the setting of a chronic culture-negative infectious endocarditis with *Streptococcus pyogenes* and recovered well with no further cardioembolic phenomenon.

**Conclusion:**

This case serves to highlight the importance of having a higher index of suspicion in any cardiac prosthesis patient for endocarditis when presenting with symptoms such as recurrent stroke, arrhythmias, and abnormal cardiac lab work. It also demonstrates the need for appropriate imaging with transthoracic echocardiography followed by transesophageal echocardiography and reviews surgical indications to diagnose and treat culture-negative endocarditis.

**Supplementary Information:**

The online version contains supplementary material available at 10.1186/s13256-024-04623-y.

## Introduction

This case highlights several complications of late-presenting culture-negative *Streptococcus pyogenes* endocarditis of a previously repaired mitral valve with an annuloplasty ring 3 years prior to presentation and complicated by recurrent cardioembolic strokes. *S. pyogenes* is a rare cause of infectious endocarditis with around 20 cases reported in the clinical literature in adults from 1940 to 2019 [1]. There is little published information on these specific cases and how to develop a diagnosis and treatment plan. Further, as culture-negative endocarditis can be a difficult diagnosis to establish, this case serves to highlight the need for a higher index of suspicion of endocarditis in any cardiac prosthesis patient presenting with symptoms such as recurrent stroke, arrhythmias, and abnormal cardiac lab work. It further demonstrates the need to perform appropriate imaging with transthoracic echocardiography (TTE) followed by transesophageal echocardiography (TEE), computed tomography angiography (CTA), or positron emission tomography (PET). Finally, this case highlights the treatment algorithm between medical management and when surgery is indicated.

## Case presentation

The patient is a 66-year-old Caucasian woman who presented to the emergency room for a tachycardia of 160 beats per minute (bpm) incidentally noticed on her smartwatch, although she was asymptomatic. Prior medical history is significant for uncomplicated robotic surgical mitral valve repair and annuloplasty for degenerative mitral regurgitation and prolapse of the posterior leaflet, with concomitant left atrial appendage clipping 3 years before the current presentation, right vertebral artery occlusion, fibromuscular dysplasia, hyperlipidemia, and depression. The patient takes aspirin 81 mg daily, ticagrelor 90 mg twice per day, atorvastatin 80 mg daily, bupropion 300 mg daily, and fluoxetine 40 mg daily. Six months prior to current presentation, she developed a right vertebral artery occlusion stroke affecting the posterior inferior cerebellar artery (PICA) territory attributed to fibromuscular dysplasia (with normal TTE performed); then, 5 months after, she developed another left parietal stroke while the patient was on dual antiplatelet therapy (DAPT) with no repeat TTE at that time. There was no prior history of arrhythmia.

**Fig. 1:**
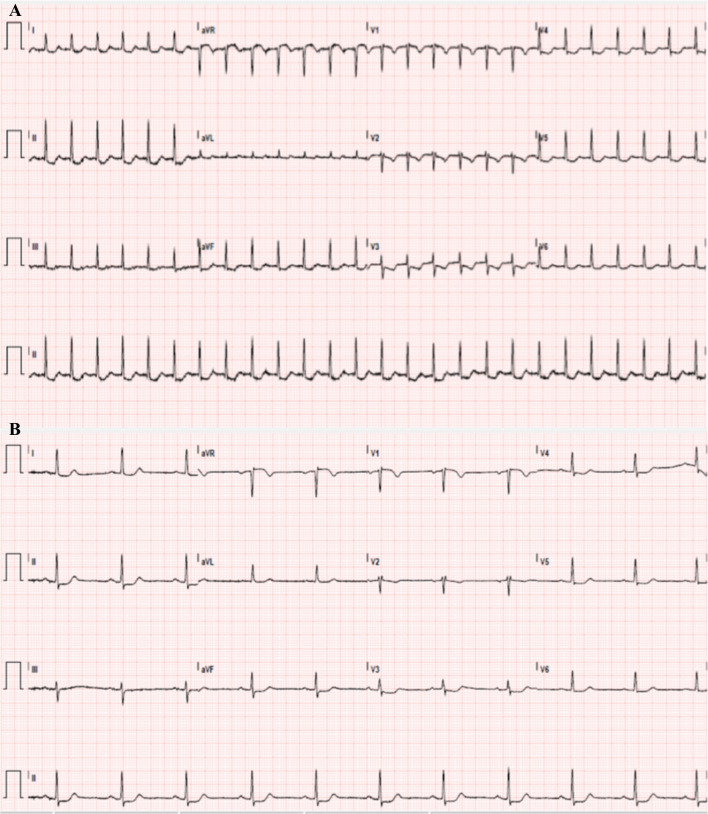
Initial electrocardiogram. **A** Shows Supraventricular Tachycardia with **B** Normal Sinus Rhythm with ST segment depressions in leads II and V3–V5

Initial electrocardiogram (ECG) showed supraventricular tachycardia (SVT) that spontaneously resolved in the emergency room to sinus rhythm with ST segment depressions in leads II and V3–V5 (Fig. 1). On physical examination, the patient had a heart rate of 64 and blood pressure of 121/63, was afebrile, and was saturating at 99% on room air. The patient had a grade II/VI systolic murmur auscultated at the left lower sternal border. There were no skin findings. High-sensitivity troponin (hs-T) was positive at 86 ng/L and rose to 777 ng/L (normal range 0–20 ng/L). She was admitted for evaluation of the ST segment depressions on her ECG and elevated hs-T. The first night of admission, she developed a new right lower facial droop and possible mild expressive aphasia, which were only transient and resolved quickly.

**Fig. 2:**
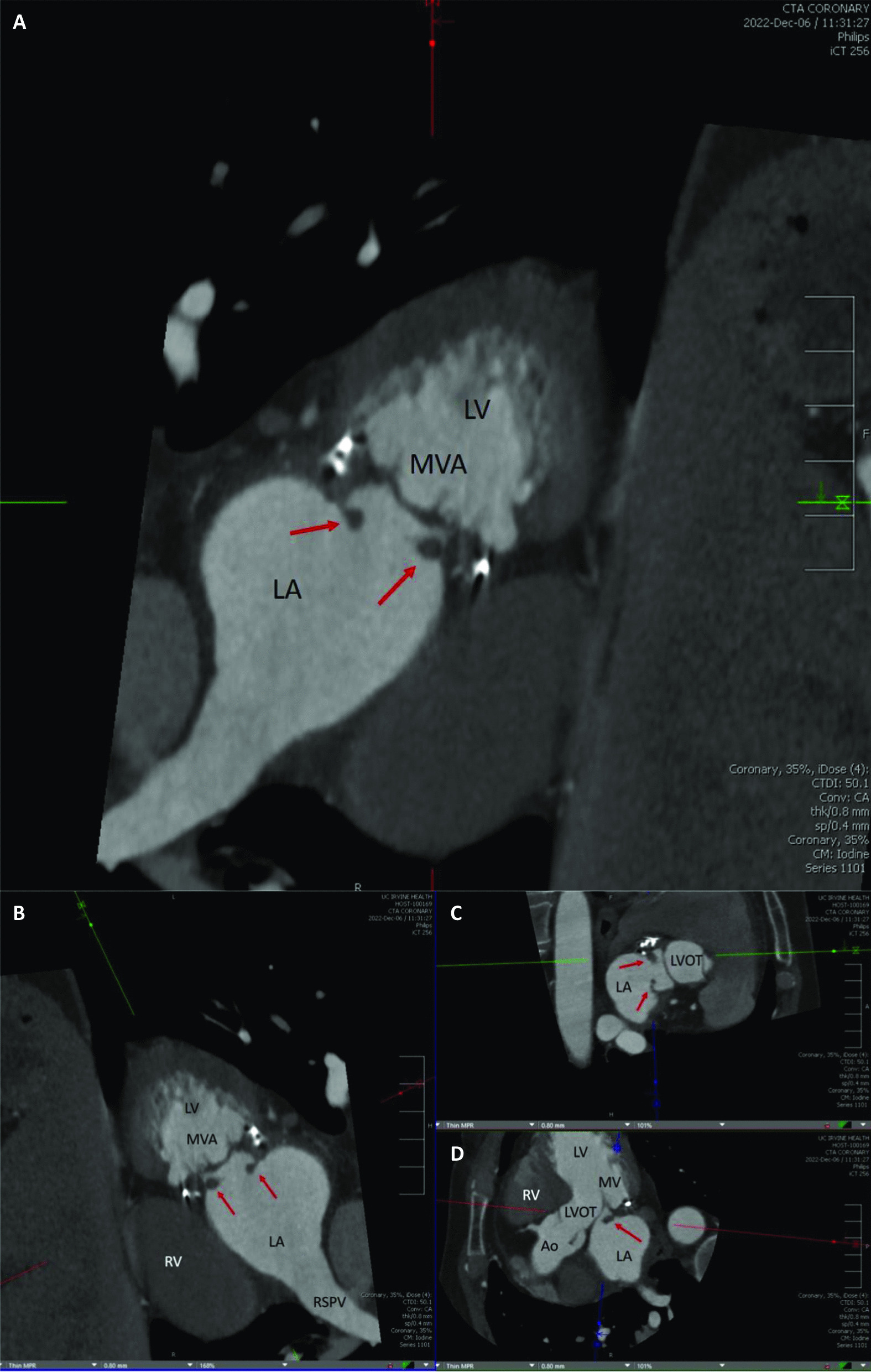
Computed tomography coronary angiogram. **A** Computed tomography coronary angiography of the mitral valve annulus demonstrating two filling defects, representing endocarditis (red arrows). **B** A multiplanar computed tomography coronary angiogram array showing filling defects in the left atrium. The labeled structures include the left ventricle, right ventricle, right superior pulmonary vein, aortic root, left ventricular outflow tract, and coaptation of the mitral valve leaflets. **C**, **D** Provide additional views

**Fig. 3:**
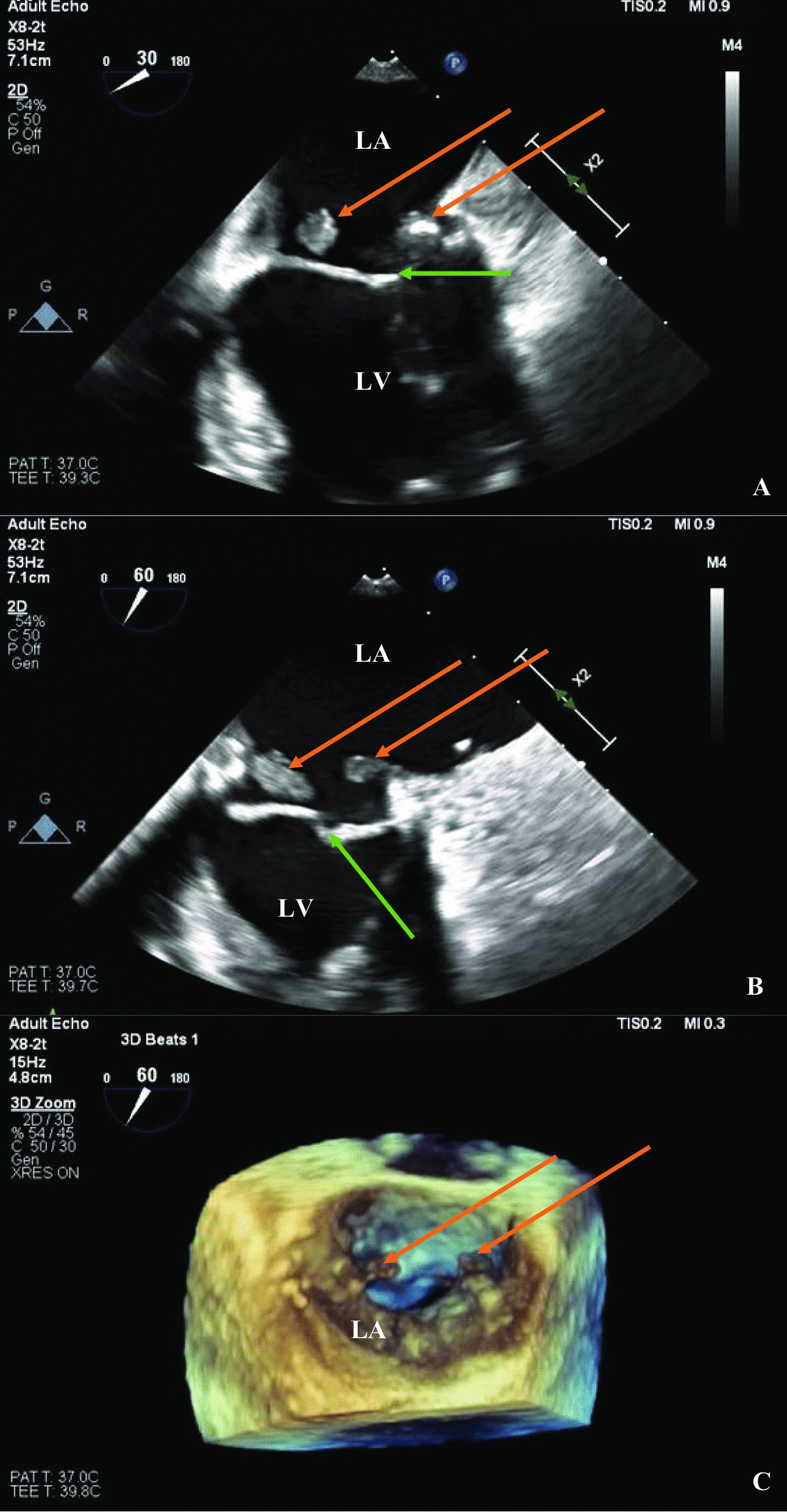
Transesophageal echocardiography images. **A**, **B** Transesophageal echocardiography at the mitral valve commissural view at 30° and 60°, respectively, with the two mitral annular masses shown by orange arrows and the mitral valve with green arrows. **C** Transesophageal echocardiography from the mid-esophageal short axis position revealing the three-dimensional “surgeon’s view.” The masses again shown with orange arrows. The left ventricle is denoted LV

The differential diagnosis included a new ischemic stroke, transient ischemic attack (TIA), recrudescence, cardioembolic stroke, hemorrhagic stroke, occult arrhythmia, interatrial septal abnormality, non-ST segment elevation myocardial infarction (NSTEMI), endocarditis with septic emboli, and toxic metabolic encephalopathy. TTE revealed preserved left ventricular systolic function (LVEF 60%), normal right ventricular systolic function, normal chamber sizes, and no wall motion abnormalities. The mitral valve was difficult to visualize, and there was evidence of prior repair and mild regurgitation. A cardiac coronary computed tomography (CT) incidentally revealed two filling defects attached to the mitral valve anulus (Fig. 2) and a coronary calcium score of 11. Magnetic resonance imaging (MRI) of the brain showed a subacute infarction of the left parietal corona radiata. TEE demonstrated two small mobile masses measuring 0.99 cm × 0.44 cm and 0.66 cm × 0.42 cm adherent to the annuloplasty ring, atrial septal aneurysm without a patent foramen ovale, a left atrial appendage closure, mitral valve repair with P2 resection, and insertion of a number 35 ATS band (Fig. 3, videos 1–2). Blood cultures were negative and a coronary angiogram confirmed nonobstructive coronary artery disease.

The patient was placed on empiric ceftriaxone, then scheduled for urgent redo mitral valve surgical intervention. This was the patient’s first sternotomy since the first surgery was a robotic repair. She underwent removal of two intra-atrial masses, explantation of the annuloplasty ring, and repeat repair and reconstruction of the mitral valve annulus using bovine pericardial patch 3 days after TEE demonstrated possible vegetations. Intra-operatively visualization suggested evidence of chronic infection of the annuloplasty ring. Pathology of the vegetative masses eventually grew *S. pyogenes*, confirming infectious vegetations. Her postoperative course was complicated by mild cardiogenic shock and junctional bradycardia briefly requiring dobutamine and norepinephrine drips and temporary pacemaker placement. These were attributed to postsurgical complications and were all successfully weaned by postoperative day 6 with diuresis. She spontaneously converted back to normal sinus rhythm on postoperative day 5. There were no further episodes of SVT. Intravenous (IV) ceftriaxone for 6 weeks for infective endocarditis was prescribed.

**Fig. 4:**
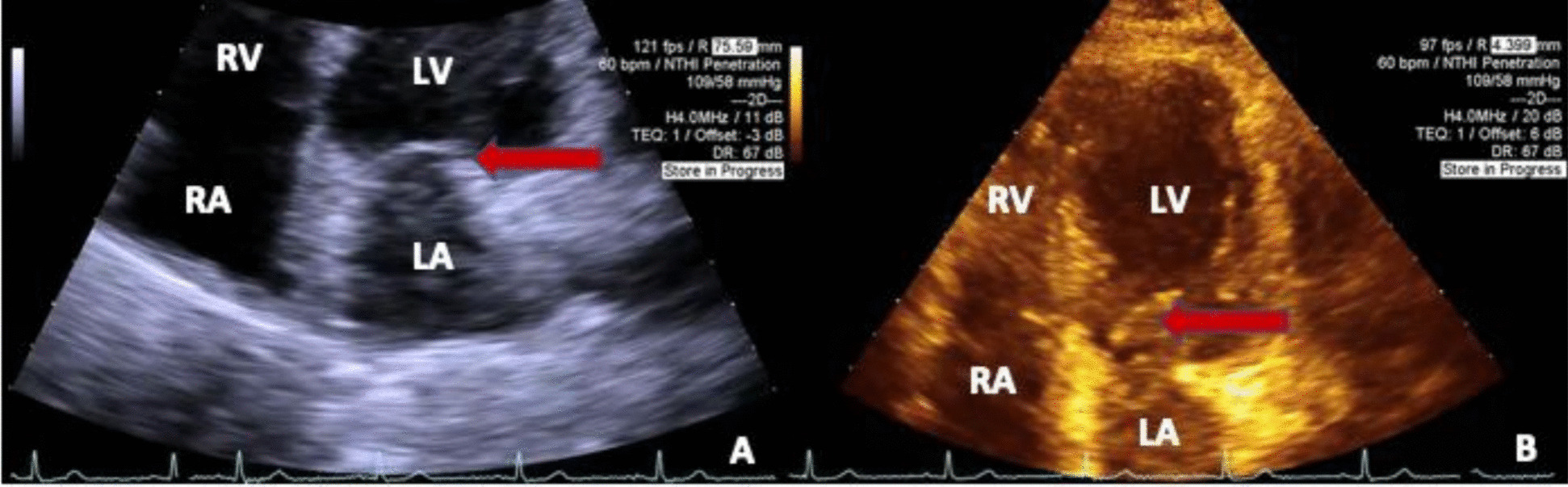
Transthoracic echocardiography images from post hospital follow up. **A** An apical four chamber view of the heart with the left atrium, left ventricle, right atrium, right ventricle, and the mitral valve indicated by the red arrow. **B** Another apical four chamber view with the same labels, again with the mitral valve indicated by the red arrow. The two intra-atrial masses are no longer visualized

After discharge, the patient recovered well and had no further recurrence of stroke or arrhythmia at 3 months. Repeat TTE demonstrated no further mitral valve pathology (Fig. 4). For an overview of the case presentation, please see the timeline in the supplemental materials.

## Discussion

This case highlights several important issues related to mitral valve repair, recurrent cardioembolic cerebrovascular accidents (CVA), and rare bacteria causing endocarditis. *S. pyogenes* is a rare cause of infectious endocarditis with around 20 cases in literature in adults since 1940 up to 2019 [1]. *S. pyogenes* is a part of normal skin flora and usually causes cutaneous infections such as cellulitis, impetigo, and pharyngitis. It may produce toxins that result in toxic shock syndrome and scarlet fever [1]. The mitral valve may be exposed to bacterial infection through bacteremia. Blood stream infections with *S. pyogenes* are usually attributable to a primary infection; however, in several cases, a primary predisposing infection was not identified (26% in one case series) [1]. Our patient had no obvious predisposing infection, no prior dental procedures, and no IV drug use history. This raises concern that her endocarditis might even be the result of an indolent infection that may have happened during the original mitral valve repair. While this cannot be confirmed and would be highly unusual, it could present as chronic infection with abscess formation seen intraoperatively and multiple strokes over several months despite remaining afebrile with negative cultures [2]. Such a presentation would be unusual as direct contamination with abscess formation is usually associated with an early infectious endocarditis post repair, but our patient’s presentation was delayed until her first CVA more than 2 years after the initial surgery [2]. It may also have resulted from another undetected bacterial infection through transient bacteremia. Additionally, the initial stroke was attributed to fibromuscular dysplasia and TTE was negative with no additional imaging requested. After the consequent stroke, a TTE was not performed, which resulted in a delay in diagnosis. Regarding mitral valve infectious endocarditis, embolic phenomenon is seen in up to 51% of reported cases with the central nervous system (CNS) and skin being the primary sites of emboli [1]. Another case series showed that stroke occurred in 9.6% of infectious endocarditis cases and that mitral valve endocarditis had a greater stroke incidence than other sites of endocarditis [3]. This is in keeping with our patient’s presentation with mitral and CNS involvement. We observed that, despite *S. pyogenes* being readily culturable, our patient’s cultures all remained negative [4]. It is unclear why this is, but it may be related to transient bacteremia or low overall bacterial titers, which is rapidly cleared and missed once cultures are drawn.

Diagnosis of culture-negative endocarditis is challenging and requires a high level of suspicion and empiric therapy may include vancomycin or ampicillin/sulbactam along with an aminoglycoside [5]. Rifampin can be added if there is a cardiac prosthesis [5]. Definitive therapy is dependent on culture outcomes [5]. Our patient was started on vancomycin, gentamicin, and rifampin, then transitioned to ceftriaxone monotherapy by our infectious disease team recommendations. Generally, *S. pyogenes* endocarditis can be treated with penicillin G; however, our patient developed a fever after starting penicillin G, so she was continued on ceftriaxone instead [1].

Echocardiography does not always show endocarditis reliably, and recently, there has been evaluation of other imaging modalities including CT, MRI, and nuclear imaging [6]. A review article suggests that for left-sided endocarditis of the native valve, both TTE and TEE should be performed first to assess the anatomy and hemodynamic involvement [6]. If the TTE is negative, TEE should be done as it has higher sensitivity for vegetations (90–100%) as compared with TTE (40–63%) [6]. If both are negative, they should be repeated 1 week later if suspicion remains [6]. Providers can consider CTA if there is concern for paravalvular infection with extension, preoperative risk stratification, procedure planning, and to look for coronary disease [6]. Positron emission tomography (PET) may also be considered as it would provide metabolic suggestion of abscesses, but there is no evidence for this in clinical practice yet [6]. These modalities can be used in combination with one another to maximize diagnostic yield and prepare for interventions [6]. In our case, the patient’s initial CVA workup did not include TTE, and when it was performed, TEE did not follow it. This resulted in a delay in diagnosis and a repeat CVA. Ironically, once she was hospitalized for an asymptomatic SVT, she had a recurrent TIA, and then she underwent several of these imaging techniques, which demonstrated the vegetations and allowed procedural planning and subsequent surgery.

According to a consensus recommendation by the European Society of Cardiology, endocarditis prophylaxis in heart valve surgery should include preoperative nasal treatment for *S. aureus* carriers, skin preparation, avoidance of hematoma and hemopericardium formation, prophylactic antibiotics directed primarily against staphylococcus species for 48 h or until chest drains are removed, and careful maintenance of IVs and urinary catheters [7]. Prevention of late prosthetic endocarditis includes education on oral hygiene, regular dental care, and antibiotic prophylaxis [7]. Antibiotic prophylaxis should be directed based on what type of procedure the patient will undergo, such as against streptococcal species for dental procedures (amoxicillin) or against *Enterococcus faecalis* for gastrointestinal or genitourinary procedures (ampicillin and gentamycin) [7]. This patient had no procedures that warranted such prophylaxis, and it is unclear what preprocedure prophylaxis she received prior to her mitral valve repair.

The guidelines by the American Association of Thoracic Surgery in 2016 recommend surgery in prosthetic valve endocarditis when there is relapsing infection without another source (class IIa), recurrent emboli and persistent vegetations despite appropriate antibiotic therapy (class IIa), or when there are mobile vegetations greater than 10 mm in length with embolic phenomena (class IIb) [8]. If indicated, they recommend surgery within days (class I) [8]. Further, a minimum multidisciplinary team consisting of cardiology, infectious disease, and cardiac surgery is recommended [8]. In our case, the patient was treated with a multidisciplinary team consisting of infectious disease, cardiology, cardiothoracic surgery, and neurology. Fortunately, our patient was spared mitral replacement, successfully underwent appropriate surgical repair, and recovered well.

## Conclusion

This case highlights the difficulty of diagnosing culture-negative endocarditis and underscores the need for clinicians to consider rare causes of endocarditis including *S. pyogenes* and the different imaging required to obtain the diagnosis. It also highlights late presenting complications of mitral valve annuloplasty including late presenting endocarditis and emphasizes the differential diagnosis of cardioembolic or septic embolic causes of stroke, particularly in patients with recurrent episodes of strokes and predisposing prosthetic heart implants including annuloplasty rings. It clearly demonstrates that TTE may not be sufficient to diagnose vegetations or small intracardiac masses and TEE should be obtained as an adjunct with CTA when endocarditis is suspected. Finally, it reiterates appropriate empiric antibiotic therapy, the indications for endocarditis prophylaxis, and different algorithms of surgical intervention and management of complicated endocarditis.

## Learning objectives

1. To be able to make a diagnosis of late presenting infectious endocarditis following mitral valve repair.
2. To recognize the link between cardioembolic stroke phenomenon and possible endocarditis.
3. To review a rare cause of infectious endocarditis.
4. To review imaging concerns for infectious endocarditis.
5. To review recommendations for valve surgery endocarditis prophylaxis.
6. To review surgical guidelines for prosthetic valve endocarditis.

### Supplementary Information

Supplementary Material 1. Video of the TEE mitral valve commissural view. It reveals two vegetations as pedunculated, mobile echo densities on either side of the bio-prosthetic mitral valve annulus.

Supplementary Material 2. Video of 3D TEE of the mitral valve annulus in the surgeon’s view. The two valvular vegetations are seen on the posterior rim of the bio-prosthetic valve annulus.

## Data Availability

Not applicable.

## Abbreviations

TTE: Transthoracic echocardiography
TEE: Transesophageal echocardiography
CTA: Computed tomography angiography
PET: Positron emission tomography
PICA: Posterior inferior cerebellar artery
DAPT: Dual antiplatelet therapy
BMP: Beats per minute
SVT: Supraventricular tachycardia
hs-T: High sensitivity troponin
TIA: Transient ischemic attack
NSTEMI: Non-ST elevation myocardial infarction
LVEF: Left ventricular ejection fraction
CT: Computed tomography
MRI: Magnetic resonance imaging
IV: Intravenous
CVA: Cerebrovascular accident
CNS: Central nervous system
